# Understanding perceptions of involving community pharmacy within an integrated care model: a qualitative study

**DOI:** 10.1186/s12913-020-05237-y

**Published:** 2020-05-11

**Authors:** Jennifer D. Lake, Zahava R. S. Rosenberg-Yunger, Katie N. Dainty, Teagan Rolf von den Baumen, Amanda C. Everall, Sara J. T. Guilcher

**Affiliations:** 1 Leslie Dan Faculty of Pharmacy, University of Toronto, 144 College Street, Toronto, ON M5S 3M2 Canada; 2 Institute of Health, Policy Management and Evaluation, 155 College Street, Toronto, ON M5T 1P8 Canada; 3 Faculty of Medicine, University of Toronto, 1 Kings College Circle, Toronto, ON M5S 1A8 Canada; 4 Ted Rogers School of Management, School of Health Services Management, Ryerson University, 350 Victoria Street, Toronto, ON M5B2K3 Canada; 5 North York General Hospital, 4001 Leslie St, Toronto, ON M2K 1E1 Canada

**Keywords:** Community pharmacy, Integrated care model, Integration, Multimorbidity, Health services, Primary care teams, Organizations, Qualitative

## Abstract

**Background:**

Over the past several years, there has been more emphasis on integration within health care. Community pharmacy is often under-represented within integrated care models. This study explored stakeholder perceptions and enablers of including community pharmacy within an integrated care model.

**Methods:**

A qualitative study was undertaken. Participants were recruited through professional networks and social media, as well as snowball recruitment from other participants. They included community pharmacists, clinicians, and decision-makers working in Ontario, Canada. Data were collected using telephone interviews completed with a semi-structured interview guide based on Consolidated Framework for Implementation Research from June to September 2018. Data were analysed inductively and deductively following the Qualitative Analysis Guide of Leuven. An additional theoretical framework (Rainbow Model of Integrated Care) was used to categorize enablers.

**Results:**

Twenty-two participants were interviewed including nine pharmacists, seven clinicians, and six decision-makers. Three key themes were identified: 1) Positive value of including pharmacy in integrated care models; 2) One model does not fit all; and 3) Conflict of interest. Four key enablers were identified reflecting functional and normative factors: functional - 1) remuneration, 2) technology; normative - 3) engagement, and 4) relationships. While both functional and normative factors were discussed, the latter seemed to be more important to facilitate the inclusion of community pharmacy. Many participants characterized community pharmacists’ lack of skills or confidence to provide patient care.

**Conclusions:**

This study confirms previously known views about concerns with community pharmacy’s conflict of interest. However, discordant perceptions of conflict of interest and negative perceptions about capabilities of community pharmacy need to be addressed for successful integration. Normative enablers, such as culture, are likely important for organizational integration and require additional inquiry.

## Background

Over the past several years, there has been more emphasis on integration within health care [1–8]. In a 2016 World Health Organization report on integrated care, “integration is a coherent set of methods and models on the funding, administrative, organizational, service delivery, and clinical levels designed to create connectivity, alignment and collaboration within and between the cure and care sectors.” [8] The complexity of medical and social care often requires effective collaboration across sectors for optimal delivery, outcomes, care experiences, and efficiency [8]. Integrated care can be delivered by various types of models which focus on individuals (e.g., case coordination), specific disease states (e.g., chronic disease management), or populations (e.g., Veterans Health Administration) [8]. Integrated care models aim to facilitate the appropriate delivery of health care services and overcome fragmentation between providers by coordinating care of high-risk patients and/or those with multiple conditions [1, 8]. There is growing recognition that integrating care across health sectors can improve patient outcomes, especially among those with complex health and social needs [9–11].

Interestingly, even though target populations for integrated care models often have complex medical conditions and may take multiple medications (known as polypharmacy) [12], integrated care models have limited focus on the inclusion of community pharmacy. Community pharmacy is the health care setting where pharmacists provide professional services such as counseling and medication supply [13]. In Canada, approximately 70% of all pharmacists work in community pharmacy [14], and could potentially provide more services to persons with complex needs within an integrated model. Previous research has demonstrated benefits from community pharmacy involvement with patients in general and in integrated care models [15, 16]. A recent article by Lapointe-Shaw and colleagues showed medication reviews by community pharmacists in Ontario, Canada were associated with a reduced risk of 30-day mortality or readmission [16]. Previous research has shown that patients receiving care from community pharmacy within integrated care models experienced improved asthma control [17], diabetes care (e.g., reductions in hemoglobin A1C) [18, 19], and use of sedative/ anticholinergic drugs in older patients [20].

A potential barrier to community pharmacy’s inclusion with integrated care models is related to their funding. This concern is especially salient in publicly funded systems where most organizations within the integrated care models are publicly funded [21], whereas community pharmacy is often funded through multiple private and public streams. In earlier studies of integration at individual levels, physicians often viewed community pharmacy as solely a commercial entity [22–27], were unaware of pharmacists’ abilities to provide direct patient care [23, 28], and had perceptions about increased conflict of interest due to funding being tied to medication dispensing [22–25]. It is unknown how these individual perceptions may affect organizational integration.

Given the gap of including community pharmacy within integrated care models, we sought to understand stakeholder perceptions of including community pharmacy within an integrated care model.

## Methods

### Study design and settings

A qualitative approach was selected, using interpretive descriptive analysis, due to the exploratory nature of the inquiry [29]. Qualitative methodology was selected to explore key stakeholder perceptions of how community pharmacy may be linked with an integrated care model and what enables this linkage.

In 2012, an integrated care model called the Health Links Approach to Care was instituted in Ontario, Canada (most populous province) [21]. As per Goodwin (2016), the Health Links Approach to Care was an example of integration at an organizational level both horizontally and vertically [30]. Specifically, the integration was designed to create care networks (horizontal integration) [30], as well as integration across different health care settings (vertical integration) [30] for a subgroup of people with complex health and social needs. The target population within the Health Links Approach to Care was "high cost users", defined as Ontarians who have the highest health care expenditures (“top 5%”) in publicly funded services during the previous fiscal year [31, 32]. Research showed that these patients accounted for more than 60% of all health care spending [32] and used an average of 13 medications [15]. The rationale for targeting these patients within the Health Links Approach to Care was to improve quality of care and to maintain or reduce health care costs and spending [33–35].

The Health Links Approach to Care was implemented across Ontario, Canada. The Ministry of Health and Long-Term Care adopted a “low-rules” approach to implementation. Each site was predicated on organizations working within their geographical area to design local solutions, network health care and social services, and provide care to persons with high health care costs [33]. The only organizational parameters mandated by the Ministry of Health and Long-Term Care were the inclusion of an appropriate mix of health care providers, including at least 65% of the family physicians in the geographical area, and the ability to track and manage persons with high-cost [33]. Most integrated care models, such as the Health Links Approach to Care, included publicly available services such as physicians, hospitals, home care, and social services in their appropriate mix [33]. This integrated care model shares similarities with American Accountable Care Organizations and the English Virtual Ward model [33].

### Study population

The study population were community pharmacists, clinicians and decision-makers from an integrated care model, and decision-makers from the health regions. At the time of the study, Ontario had 14 distinct health regions. Recruitment strategies included social media (Twitter®) advertisements, researchers’ professional networks, and outreach from related organizations such as Ontario Health (Quality; formerly Health Quality Ontario) and the Ontario Pharmacists Association. Participants were screened upon initial contact for their professional role (i.e., pharmacists, other clinicians, decision-makers) and health region to maximize variation in these two aspects. Given that the Health Links Approach to Care had been implemented differently in each region, we specifically sought to capture the experiences in different geographical areas. Pharmacists were eligible if they were licensed to practice in the province of Ontario. Clinicians were defined as any health care professional employed within an integrated care model, such as nurses, physicians, and social workers. Decision-makers were those individuals with formal leadership or management roles in either an integrated care model or a local health region. Participants were required to speak and understand English to participate. Interviews were scheduled directly by the study coordinator (TRVDB) via email or telephone. Follow-up was done twice at weekly intervals as necessary.

### Data collection

Data were exclusively collected through telephone interviews (June to September 2018). Purposeful sampling with additional snowball sampling through included participants was also used [36]. We recruited until participant saturation, which was estimated to be 25 participants [37, 38]. All participants provided informed verbal consent prior to the interviews. All interviews were audio-recorded and fully transcribed by a professional transcriptionist. Interviews were completed, reviewed, cleaned, and de-identified by two members of the study team (ACE and TRVDB). Team members directly involved in recruitment, conducting the interviews, and de-identification were trained in qualitative methods, and mentored by more senior researchers (SJTG and JDL). The interviewers created a short reflexive report after each interview including any questions or thoughts about the interviews, which were reviewed with the study team.

A semi-structured interview guide was created using a combination of expert opinions, theoretical concepts, and constructs from the Consolidated Framework for Implementation Research (CFIR) [39, 40]. CFIR was initially selected for its strong theoretical basis, the wide range of constructs included, and its use in a diverse array of scenarios. The interview guide questions were open-ended to gather perceptions about linking community pharmacy with integrated care models (e.g., the Health Links Approach to Care) and potential enablers. The interview guide was piloted to establish acceptability and ensure usability. Pilot interviewees were conducted with individuals from each professional group who did not meet our inclusion criteria but were able to speak to the topic.

### Data analysis

Data collection and analysis occurred simultaneously. Analysis was conducted using principles of the Qualitative Analysis Guide of Leuven (QUAGOL) [29]. Coding was done iteratively throughout data collection with multiple team members involved in creation of the initial codebook. The codebook was created from conceptual interview schemes, which were informed by sensitizing concepts from CFIR [39] (i.e., deductive) and from the raw data (i.e., inductive). Analysis of coded data was done by two of the team members, both trained in qualitative methods with one being a senior researcher (SJTG) and the other currently completing a PhD using similar methods (JDL). Identified themes were compared throughout analysis, and each team member had opportunities to comment and reflect on the data’s consistency and comprehensiveness [41]. Themes were further distilled by comparing them to each other to come to overarching themes, these were discussed and modified as necessary.

Based on interim findings, an additional conceptual framework was adopted, the Rainbow Model of Integrated Care, to categorize factors associated with integration in more detail [42–44]. Using the Rainbow Model of Care, a conceptual framework of integration, enablers were further subdivided into functional (the technical aspects), and normative (the cultural aspects) enablers [42]. Functional enablers are the key support functions of primary processes (e.g., financial, information systems) [43]. Normative enablers can be viewed as the “soft” cultural aspects of integration defined as a common reference of mission, vision, value, and culture [42, 43]. The data were managed using NVivo 11 software [45].

Credibility of the results was maintained in several ways [41]. Rigor was enhanced through the involvement of participants with maximal variation, standardization of interview questions, and the use of two trained interviewers (ACE, TRVDB). To maintain validity of the data, the main analyst kept a research diary throughout the duration to document personal reactions or events, and to maintain tracks between the data collected and final thematic results. Peer debriefing was used throughout all stages of analysis and writing to ensure agreement of interpretations. Interview quotes were presented in full detail to maintain their context.

Our study received approval from the University of Toronto Research Ethics Board (REB 30695) and Ryerson Research Ethics Board (REB 2019–129).

## Results

A total of 22 participants from six different health regions in Ontario, Canada participated in an interview. Table 1 outlines characteristics of the participants.

**Table 1:** Participants Characteristics (*n* = 22) *

	Pharmacists (*n* = 9)	Clinicians (*n* = 7)	Decision-makers (*n* = 6)
Female	6	7	6
Years in Practice, (median-range)	10 (5–37)	11 (10–30)	N/A
Years in current role, (median-range)	N/A	N/A	5 (1–8)
Health Regions			
A	3	-	-
B	2	-	-
C	2	4	2
D	-	3	3
E	1	-	-
F	1	-	1

* Note: Decision-makers described their years in current roles as they were not always clinicians and therefore did not have a specific number of years in practice resulting in a lower number compared to pharmacists and clinicians

Three key themes were identified: 1) Positive value of including pharmacy in integrated care models; 2) One model does not fit all; and 3) Conflict of interest. Four enablers were identified and were characterized using the Rainbow Model of Integrated Care’s functional (remuneration and technology) and normative factors (engagement and relationships/ trust) [42, 43]. Each of these themes will be discussed in more detail below.

### Positive value of including pharmacy in integrated care models

There was unequivocal support from all participants about pharmacist involvement with in integrated care models. Participants recognized community pharmacists were accessible, had medication expertise, and had knowledge of patients’ medication history. Participants spoke about the role pharmacists can play in improving adherence among those with frequent medication needs, transitions of care, and daily dispensing.

*The bulk of patients are not in team models so, the fact that pharmacists are a general availability, are a universal service in this province. […] it’s just really important. (Clinician, C04).*

*I see a pharmacist playing a key role in making sure the meds piece is looked at and sorted and then communicated to everybody […] I see them playing a role in smoother transition. (Decision-maker, D05).*

*It would certainly be helpful to have a drug expert at the table. (Pharmacist, P05).*

Additionally, pharmacist participants discussed how integration may bring value to the contributions they make or could make by providing clinical services for patients within an integrated care model.

*They can talk adherence. They can talk to what they tried in the past. (Pharmacist, P03).*

*It’s beneficial because it puts community pharmacists into that care team, which we’ve always been there, we just haven’t been recognized as a resource. (Pharmacist, P04).*

### One model does not fit all

Participants were not in agreement about how integration should occur. Pharmacists accepted the idea of organizational integration but the majority of clinician and decision-maker participants conceptualized a different model. Clinicians and decision-makers suggested hiring a pharmacist directly into the integrated care model (i.e., embedded) to work with their patients and serve as a liaison to other services.

*I hope later that I [can] have a pharmacist in my team, it’s easier to refer within the team. (Decision-maker, D03).*

*Again, there’s something to be said about physically being on-site […] the huge value of being able to do that warm hand-off and having a pharmacist physically on the same site and integrated with [the team]. Community based pharmacists, again, if they’re working on a different site, it doesn’t facilitate the collaboration as nicely as when they’re physically co-located. (Decision-maker, D05).*

One reason for the preference of an embedded model was that the pharmacist could act as an intermediary between those in the integrated care model and the community pharmacy.

*Wouldn’t it be cool if there was [an embedded] pharmacist that formed the relationship and was able to liaise with the patients’ individual [community] pharmacist. (Clinician, C04).*

### Perceptions of conflict of interest

All participants were provided with an opportunity to comment on perceptions of community pharmacy’s potential for conflict of interest. When asked directly, most participants either denied conflict of interest concerns or a dismissed the significance of conflict of interest by suggesting such conflict would be like other health care professionals (e.g., physicians). However, participants often indirectly made references to potential conflicts of interest when discussing concerns about community pharmacy’s business practices.

*I don’t know the proper word to use. But like kickbacks or incentives from pharma or drug companies. We have to be careful on that because we don’t want to promote a model that is medication-driven and […] over-prescribe or recommend specific drugs. I think we’ve got to be careful about that. (Decision-maker, D05).*

*I think there needs to be policy and billing changes […] I don’t necessarily say that all of it’s just simply for profit. It’s just if you run a business, you run a business and so the only way you can avoid these drivers are if you link these business drivers with clinical outcomes. (Clinician, C03).*

This contradiction between participants providing an answer when directly asked about conflict of interest, but also expressing an opposing view speaks to the potential unconscious bias that may exist against community pharmacy. Pharmacist participants seemed acutely aware of this perception and suggested it was common.

*There’s a presumptuous concept that because community pharmacists are for profit, they’re not your normal care provider, because all the other [integrated care model] providers are not-for-profit, or they are salaried by non-for-profit agencies […] community pharmacists need to kind of break down that barrier that although we are for profit, we’re still able to provide a service that’s missing as part of the team. (Pharmacist, P09).*

Pharmacists also identified the importance of raising awareness about the reality of conflict of interest and having conversations about these concerns.

*I think it is a very real misconception […] instead of just letting it be the elephant in the room, an unspoken thing that everybody is thinking to actually talk through that and address it head on to dispel that myth. (Pharmacist, P02).*

#### Enablers: functional and normative

##### Functional enablers

Participants commented on both types of enabling factors identified in the Rainbow Model of Integrated Care, functional and normative. The main functional enablers were related to remuneration of community pharmacy’s services and technology. Participants described the need to remunerate community pharmacists for time spent on clinical non-dispensing services (e.g., communicating with other members of the health care team), which were viewed as important for integration of care.

If *there was a compensation model. It would be a much easier sell. Because right now it’s done out of the goodness of your heart because it’s really difficult to bill for it. (Pharmacist, P04).*

*I mean we always say remuneration […] that would really help pharmacists spend that extra time on the phone or researching something for a physician. (Clinician, C04).*

Participants also spoke about the need for an electronic health or medical record (EHR/ EMR) to facilitate access to information, which would ultimately facilitate integration of care.

*I think the electronic health record is going to make a big difference for linking everyone together and maybe that will facilitate a lot of these things […] having everyone hooked up to the electronic health record, including the patients, which would be a sensible access level. (Pharmacist, P05).*

##### Normative enablers

Participants described several key normative enablers including engaging community pharmacy earlier in planning and building relationships and trust between organizations. Many participants commented on how engaging community pharmacy was important, but that this was not done in a systematic nature.

*They’re a key service provider, but I don’t think we always bring them to the table to look at transition planning. And so, I think that pharmacy could be playing a bigger role in being involved at the planning level, so their role is integrated in a standardized way. (Decision-maker, D05).*

Several pharmacists described frustration in not being included in important system planning conversations with other professions, with a participant describing it as:

*It’s a pretty tough entrance point for some reason to let pharmacy into [health regions] and I don’t understand…why they are so afraid of us, or how they fail to see the extra value we are adding. (Pharmacist, P07).*

Meaningful engagement across professions can help foster relationships and trust. Most of the participants highlighted the importance of building trust for linking community pharmacy.

*There has to be a certain level of personal relationships, as well, I think there’s a personal trust that has to be built between different health care providers so that they can rely on one another in care. (Pharmacist, P06).*

*They [community pharmacist] need to develop a relationship. They need to reach out to folks that are running the [integrated care model]and the [health region], whoever those leads are and start building those relationships. (Decision-maker, D04).*

An essential factor in building relationships was participants having positive perceptions about each other’s abilities; however, positive perceptions of pharmacists' abilities were not consistent among our participants. There was negative rhetoric regarding community pharmacy’s lack of knowledge, skill, and/or ability working with complex patients, as well as their motivation to work with in integrated care models. These negative perceptions could be an underlying barrier to collaborative relationships.

*When it comes to community pharmacy, what we see in terms of them practicing to scope,versuspharmacists that are in [primary care and hospital] they’re not at the same level on the clinical side of things. (Decision-maker, D04).*

*I think the quality difference can be quite astounding between the knowledge of dispensing pharmacists in the community [and pharmacists employed in university-affiliated sites]. So, one can’t assume that everyone in the community will be at the same level. (Clinician, C03).*

*Some pharmacists just don’t want to get involved. It doesn’t matter what you change. They just want to do what they’ve always done. (Pharmacist, P01).*

## Discussion

We explored perceptions of how community pharmacy can work with in an integrated care model and what enables integration. Our results support previous research that pharmacists are valued members of teams but there can be integration difficulties [46–48]. There were important differences in our participants’ conceptualization of and preferences for integration. Several clinicians and decision-makers suggested an embedded model which entailed hiring a pharmacist instead of integrating organizationally. The proposal of an embedded model goes against the underpinnings of organizational integration. Integration between autonomous organizations allows each organization to continue governing independently while improving the quality and efficiency of health care services [43]. Although participants spoke of the importance of trust, the proposed embedded pharmacist model can be viewed as maintenance of power within the integrated care model [49–52], allowing others to direct pharmacy services versus having community pharmacy maintain and direct their own services. The proposed embedded pharmacist model may reflect a lack of confidence in community pharmacists’ skills and legitimizes oversight of the pharmacist. Thus, the proposed embedded model hinders integrating autonomous organizations due to a desire to deliver and maintain all services under one overarching organized network.

The negative perceptions of community pharmacy seemed to underlie why community pharmacy was not included in integrated care models and may have led to the suggestion of a different model of integration. Across participants there was significant negative rhetoric towards community pharmacy. Many participants, including community pharmacists, characterized community pharmacy’s lack of skills or confidence to provide patient care within an integrated care model. This negative rhetoric is an essential area for further inquiry, as a negative professional identity of community pharmacy may underlie the lack of organizational integration [53, 54]. Negative professional identities, both overt and covert, make building relationships difficult [53, 54]. Pharmacists providing negative opinions of their own profession may be more detrimental, as other people will build their perceptions of community pharmacy on the expertise of a colleague’s recommendation. If relationship building and trust are essential enablers, the professional identity of community pharmacy may be an important enabler which needs further investigation. This negative culture being a barrier to pharmacy practice change has been previously posited by Rosenthal and colleagues [55, 56], but has not been discussed with respect to integrating organizations. Since the majority of pharmacists practice in community settings, it could be an important area for further research.

We also identified an implicit perception of conflict of interest about community pharmacy’s business model among most participants. This perception of conflict could affect clinicians’ willingness to engage with community pharmacists, which would affect relationship building and ultimately trust. These concerns are consistent with current literature [22–25], which describes the professional identity of pharmacists as shopkeepers [22, 26, 27]. Professional identity is the social construct of how pharmacists and society perceive the profession [53]. These ongoing perceptions of shopkeepers and of increased conflict of interest can devalue the patient care provided by community pharmacists. Our participants denied these concerns when asked directly but reverted to these concerns when prompted or when responding to other questions during the interviews. This discordance between consciously versus unconsciously stated opinions will make it difficult to move integration forward because perceptions can only be managed when participants are aware of assumptions and biases. Social desirability and the desire to maintain harmony within working relationships may underlie this observed discordance [53]. Our findings are consistent with recent work by Altman, who discusses the discordance between increasing clinical workload and lack of delegation of clinical services due to discomfort of the retail space [26]. The ongoing culture issue of professional identity of community pharmacy and its impact on organizational integration requires further exploration.

There were a few limitations to this study. Firstly, there was a change in government and health care priorities during data collection which may have impacted the participants. These changes included government officials declaring potential cessation of the integrated care model. Participants discussed concerns regarding the viability of the current integrated care model (i.e., Health Links Approach to Care) and uncertainty about the health care system in Ontario. Secondly, representation of clinicians was only from two health regions so there may be some contexts which were over-emphasized. However, this did not seem to be the case, as we came to data saturation across the entire participant group. The nature of qualitative research means that the data may be limited in other contexts or settings; however, the richness of the data and the data saturation provides confidence of its insight into the study question. Thirdly, those who agreed to participate may be more engaged in research or hold different views than others who did not volunteer. Fourthly, we did not ask participants about their previous interprofessional education which may have been a factor that impacted their ability to collaborate, changed their perceptions of pharmacy and integration, or increased their exposure to pharmacy prior to their current work settings [57]. Finally, within interviews, there can be a concern about participants providing answers based on social desirability; however, we were able to discern discordances within responses and the negative rhetoric suggests social desirability was not a concern.

## Conclusion

Community pharmacy is the setting in which most pharmacists practice. Community pharmacists should play a role in improving patient care, especially for those with complex health and social needs. As integrated care models expand, there is a need to understand organizational integration which includes community pharmacy. This study confirms previously known views about concerns with conflict of interest. However, we identified an unwillingness to explicitly discuss perceptions about conflict of interest and other negative perceptions about capabilities of community pharmacy that need to be addressed for successful integration. These ongoing negative perceptions may impact the ability to integrate by reducing normative enablers, such as shared vision and culture. Increasing opportunities for meaningful engagement between organizations may help facilitate improved awareness of community pharmacists’ skills and foster trust.

## Data Availability

The datasets generated and/or analyzed during the current study are not publicly available due to inclusion of many direct and indirect participant identifiers, but de-identified information is available from the corresponding author on reasonable request.

## Abbreviations

CFIR: Consolidated Framework for Implementation Research
QUAGOL: Qualitative Analysis Guide of Leuven
EHR: Electronic Health Record
EMR: Electronic Medical Record
